# Economic benefits of microprocessor controlled prosthetic knees: a modeling study

**DOI:** 10.1186/s12984-018-0405-8

**Published:** 2018-09-05

**Authors:** Christine Chen, Mark Hanson, Ritika Chaturvedi, Soeren Mattke, Richard Hillestad, Harry H. Liu

**Affiliations:** 1 RAND Corporation, 1776 Main Street, Santa Monica, CA 90401 USA; 2 RAND Corporation, 1200 South Hayes Street, Arlington, VA 22202-5050 USA; 3 RAND Corporation, 20 Park Plaza, Suite 920, Boston, MA 02116 USA

**Keywords:** Amputee, Microprocessor controlled knee, Economic analysis, Incremental cost effectiveness ratio, Transfemoral amputation

## Abstract

**Background:**

Advanced prosthetic knees allow for more dynamic movements and improved quality of life, but payers have recently started questioning their value. To answer this question, the differential clinical outcomes and cost of microprocessor-controlled knees (MPK) compared to non-microprocessor controlled knees (NMPK) were assessed.

**Methods:**

We conducted a literature review of the clinical and economic impacts of prosthetic knees, convened technical expert panel meetings, and implemented a simulation model over a 10-year time period for unilateral transfemoral Medicare amputees with a Medicare Functional Classification Level of 3 and 4 using estimates from the published literature and expert input. The results are summarized as an incremental cost effectiveness ratio (ICER) from a societal perspective, i.e., the incremental cost of MPK compared to NMPK for each quality-adjusted life-year gained. All costs were adjusted to 2016 U.S. dollars and discounted using a 3% rate to the present time.

**Results:**

The results demonstrated that compared to NMPK over a 10-year time period: for every 100 persons, MPK results in 82 fewer major injurious falls, 62 fewer minor injurious falls, 16 fewer incidences of osteoarthritis, and 11 lives saved; on a per person per year basis, MPK reduces direct healthcare cost by $3676 and indirect cost by $909, but increases device acquisition and repair cost by $6287 and total cost by $1702; on a per person basis, MPK is associated with an incremental total cost of $10,604 and increases the number of life years by 0.11 and quality adjusted life years by 0.91. MPK has an ICER ratio of $11,606 per quality adjusted life year, and the economic benefits of MPK are robust in various sensitivity analyses.

**Conclusions:**

Advanced prosthetics for transfemoral amputees, specifically MPKs, are associated with improved clinical benefits compared to non-MPKs. The economic benefits of MPKs are similar to or even greater than those of other medical technologies currently reimbursed by U.S. payers.

**Electronic supplementary material:**

The online version of this article (10.1186/s12984-018-0405-8) contains supplementary material, which is available to authorized users.

## Background

There are about 185,000 amputations conducted per year in the U.S. [1]. Currently, approximately 1.9 million individuals are living with limb loss according to the Centers for Disease Control and Prevention [2], a figure expected to rise to 3.6 million by 2050 [1]. Of this number, it is estimated that 18.5 to 21.0% are transfemoral amputees [3, 4]. Transfemoral amputation, or the removal of a limb above the knee joint, is performed to remove ischemic, infected, or irreversibly damaged tissue and is generally a life-saving procedure. About 82% of transfemoral amputations are due to peripheral artery disease and/or diabetes, followed by trauma, cancer, infection, and congenital defects [5, 6].

Advanced technologies can help transfemoral amputees improve functional mobility and as a result, quality of life. A transfemoral amputee often has difficulty in regaining normal movement. For example, transfemoral amputees must use 35–65% more energy [7–10] to walk than a person with two legs due to complexities in the knee joint. Over the last decade, major technological advancements such as microprocessors, and their associated load and position sensors have catalyzed the modernization of prosthetics [11]. Such advanced prosthetic knees and feet were developed to allow for safer movements across a range of walking environments and improving user quality of life [11–13].

Despite the rapid progress in advanced technologies, our healthcare payment system, however, has not yet evolved simultaneously, treating prosthetics as commodity products and emphasizing cost-cutting rather than good value for the money. Currently, the Centers for Medicare and Medicaid Services (CMS), the Department of Veterans Affairs, and private insurance companies restrict reimbursement of prosthetics based on the Medicare Functional Classification Level, an index for classifying the functional mobility and productivity potential of individuals with lower limb loss [14, 15]. Within Medicare, amputees often have to pay about 20% of the device cost out-of-pocket when they purchase a new device; if a prosthetic device is not covered, amputees have to pay for the entire device out of pocket. Consequently, patients often choose low-cost prosthetic devices and may not realize their potential in functional mobility [16]. With increasing cost-cutting pressure in recent years, payers have shifted part of such pressure onto the prosthetics industry. For example, citing a 2011 report by the Office of Inspector General [17], the CMS issued new local coverage rules in 2015 to tighten the rules for reimbursing lower-limb prosthetics.

An open and candid dialog among stakeholders would help us strike the right balance between improving clinical outcomes and controlling healthcare cost, and this is where robust evidence should play a critical role, such as evidence for the incremental value of advanced prosthetics in comparison to conventional prosthetics. On the one hand, payers should ensure patient access to advanced technologies with proven health benefits, but on the other hand, they have the fiduciary obligation to contain ever-expanding healthcare costs. To address this, quality clinical and economic data, as well as rigorous studies, are required to demonstrate the value of prosthetics and associated services. In the absence of head-to-head clinical trial data, a modeling study was conducted to leverage existing evidence to assess the cost-effectiveness of advanced prosthetics such as microprocessor-controlled prosthetic knees compared to non-microprocessor alternatives from the societal perspective.

## Methods

The clinical and economic benefits of microprocessor-controlled prosthetic knees (MPK) were compared with those of non-microprocessor controlled prosthetic knees (NMPKs) from a societal perspective, and the results are summarized as an incremental cost-effectiveness ratio (ICER) — a commonly accepted measure for cost-effectiveness or value for money. ICER is defined as the additional resource requirements per unit of additional health gained, which is typically measured by quality-adjusted-life-years (QALY). The analysis assessed various clinical and economic endpoints, including physical function, quality of life, direct healthcare costs, and indirect costs such as the impact on caregiving expenses, transportation expenses, and work productivity.

All costs were inflated to 2016 U.S. dollars using the medical care component of the Consumer Price Index [18] and, when applicable, were converted to U.S. dollars using the exchange rate at the time the study was conducted [19]. This study was approved by RAND’s Human Subjects Protection Committee.

### Target population

The analysis focuses on the Medicare population, which includes a diversely aged patient group, because CMS not only represents the largest payer for prosthetic devices in the country but also sets the market standard for reimbursement levels against which commercial payers and the Department of Veterans Affairs often benchmark. Besides, since unilateral K3 and K4 transfemoral amputees have historically been the primary users of advanced prosthetics, they are the target population of the main analysis. Unilateral K1 and K2 transfemoral amputees were examined in the sensitivity analysis. Dobson and DeVanzo LLC provided basic characteristics of the target populations for the simulation model based on the 2011–2014 Medicare claims data (see Additional file 1: Table S1).

### Data sources

#### Literature review

PubMed, Embase, CINAHL, PsycINFO, Web of Science, and Google Scholar were searched for relevant peer-reviewed articles. References of the identified literature were manually searched for additional publications. Non-peer reviewed literature such as technical reports produced by government agencies or industry associations was also examined. For each input parameter, a range of estimates was compiled from the literature whenever possible, where the median value served as the base case in the simulation model, while the upper and lower bounds were used in the sensitivity analysis.

#### Expert panel process

An expert panel was convened to supplement the literature review, to validate the assumptions made, to ensure adequate and complete understanding of the prosthetics literature, and to ensure appropriate model development and construction. In addition, when the model parameters were not available in the published literature, experts were asked to provide estimates for such parameters. Fifteen experts were selected based on their publication record in the various topics that informed the simulation model. Telephone-based panel discussions and one-on-one interviews were conducted on an as-needed basis.

#### Cost of device acquisition

The cost of device acquisition is approximated using the current Medicare payment amount. Therefore, it does not necessarily represent the manufacturer list price. The base case value was based on expert input and the upper and lower bounds were derived from the 2016 Medicare fee schedule allowed payments [20] for the two most frequent combinations of L codes, which were identified among the new unilateral transfemoral amputees in the 2011–2014 Medicare claims data. The median of the Medicare allowed payments in the 2 years after the device fitting in the same Medicare population was used as the cost of device repair and physical therapy. Dobson & DaVanzo LLC conducted all the Medicare claims data analyses.

### Simulation model

A cohort-level Markov model [21, 22] was developed to simulate the clinical and economic outcomes for a unilateral transfemoral amputee population. This hypothetical cohort was assigned to two different treatment strategies, NMPK or MPK, with all other prosthetic components being the same. The simulation was limited to 10 years because the existing evidence comes from relatively short-term studies, meaning that longer-term predictions can be subject to large uncertainty. All health and cost outcomes were discounted to the present time using a 3% discount rate.

Because the data available from the literature permitted the conversion of only two clinical conditions, falls and osteoarthritis, into economic benefits, two modules were constructed for the model respectively. The lack of data, however, prevented the quantification of potential benefit derived from other medical conditions, such as obesity and cardiovascular diseases.

In the fall module, patients can experience three health states: fall, no fall, and death. Falls can be either medical, i.e., require medical attention, or non-medical. Medical falls can be minor, major, or fatal. Major injurious falls are associated with an admission to a medical facility. A patient may enter the “death” state from the “no fall” state due to causes other than falling. While Markov models are “memoryless,” meaning the health state at a subsequent step depends only on the state at the previous step, the model updates the annual probability of falling to simulate the effect of learning. The osteoarthritis module has three states as well: no osteoarthritis, osteoarthritis, and death. Patients can move from one state to another until the end of the 10-year time period or death.

After implementing the model, validation testing was performed to assure that the computations were done correctly, and the outputs responded as expected to changes in key parameter input values. The model was programmed in Visual Basic for Applications for Microsoft Excel and followed the modeling guidelines of the International Society for Pharmacoeconomics and Outcomes Research [23].

Model parameters were compiled from the literature review, expert consultation, and the analysis of Medicare claims data. When parameters were not available from the published literature, expert opinion was used and, if needed, assumptions were made. The model parameters, assumptions, and data sources are listed in Table 1.

**Table 1:** Model parameters, assumptions and data sources

Model parameter	Base case	Range	Data sources
Probability of falling per year			[26, 60]
MPK	26.00%	22.20–32.00%	
NMPK	82.00%	75.00–87.50%	
Proportion of medical falls	10.40%	6.20–19.60%	[61–63]
Proportion of fatal medical falls	7.00%	6.30–7.70%^a^	[64]
Proportion of major injury falls	40.00%	32.60–40.00%	[65, 66]
Proportion of minor injury falls	53.00%	53.00–60.50%	
Average number of falls per faller per year			[26, 59]
MPK	3.20	2.00–3.20	
NMPK	3.87	1.86–3.87	
Odds ratio of falling in year 4 vs. year 1	0.53	0.48–0.58^a^	[67]
Medical cost per major injurious fall	$24,844.52	$16,978.61 - $31,707.24	[65, 66, 68]
Medical cost per minor injurious fall	$1332.47	$620.69 - $6005.62	
Medical cost of fall-related death	$27,337.76	$27,337.76 - $29,578.20	[68, 69]
Caregiving expenses per person per year			[39, 70–72]
MPK	$2754.29	$2478.86 - $3029.72^a^	
NMPK	$3477.60	$3129.84 - $3825.36^a^	
Lost wages per person per year			
MPK	$1669.11	$1502.20 - $1836.02^a^	
NMPK	$2144.06	$1929.65 - $2358.47^a^	
Transportation expenses per person per year			
MPK	$463.46	$417.11 - $509.81^a^	
NMPK	$300.36	$270.32 - $330.40^a^	
Baseline prevalence of osteoarthritis (knee)			Medicare claims data 2011–2014
K1/K2	16.30%	14.67–17.93%^a^	
K3/K4	19.10%	17.19–21.01%^a^	
Probability of developing osteoarthritis per year			[34]; Expert opinion
MPK	1.50%	1.35–1.65%^a^	
NMPK	2.21%	1.99 - 2.43%^a^	
Osteoarthritis-related medical cost per year	$6639.72	$996.41 - $14,682.92	[73]
Osteoarthritis-related indirect cost per year	$1084.21	$606.89 - $1192.63	[74, 75]
Baseline mortality rate			Medicare claims data 2011–2014
K1/K2	18.00%	16.20–19.80%^a^	
K3/K4	9.31%	8.38–10.24%^a^	
Device acquisition cost in 10 years			2016 Medicare fee schedule; Medicare claims data 2011–2014; Expert opinion
MPK (plus 1 replacement)	$56,000.00	$44,750.00 - $58,118.00	
NMPK (plus 2 replacements)	$16,500.00	$7785.00 - $22,101.00	
Device repair cost per year			
MPK	$192.23	$173.01 - $211.45^a^	
NMPK	$135.95	$122.36 - $149.55^a^	
Physical therapy cost in year 1			
MPK	$1986.68	$1788.01 - $2185.35^a^	
NMPK	$1648.62	$1483.76 - $1813.48^a^	
Physical therapy cost in year 2			
MPK	$1621.68	$1459.51 - $1783.85^a^	
NMPK	$1347.47	$1212.72 - $1482.21^a^	
Health utilities			[37–40]
MPK	0.82	0.75–0.83	
NMPK	0.66	0.60–0.92	
Discount rate	3.00%	2.00–5.00%	[76]

*MPK* microprocessor-controlled knees, *NMPK* non-microprocessor controlled knees. K1-K4: Medicare Functional Classification Level 1 to 4, respectively

^a^There are no range values directly from the literature; in the sensitivity analyses, they were derived through varying the base case value up and down by 10%. The design of the studies comparing the effectiveness of MPK to NMPK: Prospective cohort study [26, 39, 59, 60]; Retrospective cohort study [37, 40]; Cross-sectional study [38])

One-way sensitivity analyses were conducted where one input parameter was changed at a time to inspect the sensitivity of model results to changes in key input parameter values as they were varied individually. Probabilistic sensitivity analyses on model inputs with 1000 replications, assuming uniform distributions for all variables, were also conducted.

## Results

### Clinical benefits

#### Physical function

A number of studies assessed the effects of advanced prosthetics by measuring biomechanical and physical performances when subjects wore NMPK and after subjects were fitted with MPK. Overall, there is strong evidence suggesting that compared to NMPK, MPK is associated with improvements in walking speed [24–26], gait symmetry [13, 27], the ability to negotiate obstacles in the environment [11, 15, 26, 28], and safety in terms of reduced stumbles and falls. However, while there is some evidence suggesting improvement in other dimensions such as energy efficiency [11, 24, 29–32] and physical activity [28, 30, 33], the results are inconclusive.

#### Falls and fall-related mortality

**Fig. 1:**
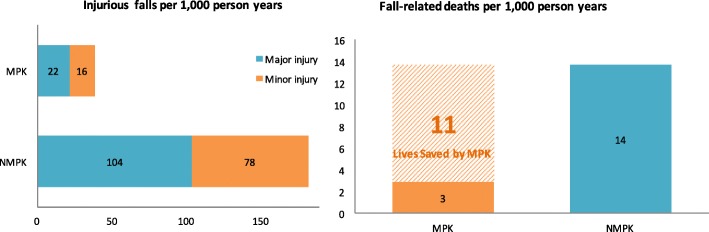
Injurious Falls and Fall-related Deaths Among MPK and NMPK Users, Note: MPK: microprocessor-controlled knees; NMPK: non-microprocessor controlled knees

Based on the simulation results, the risk of major injurious falls is reduced by 79% in MPK users compared to NMPK users, as the incidence rate decreases from 104 to 22 per 1000 person-years, and the incidence rate of minor injurious falls decreases from 78 to 16 per 1000 person-years (Fig. 1). Meanwhile, the incidence rate of fall-related deaths is 3 and 14 per 1000 person-years in MPK and NMPK users, respectively. That is, 11 lives would be saved by MPK if we observed 1000 amputees for 1 year.

#### Incidence of osteoarthritis

Kaufman and colleagues [34] observed that, compared to NMPK, MPK reduces the moment about the knee–an indirect measure of the force absorbed by the knee–of the prosthetic limb by 30%. Thus, based on expert opinion, it was assumed that MPK would reduce the onset of osteoarthritis from 20 to 14 per 100 persons in a 10-year period. Incorporating these estimates into the simulation model resulted in 16 fewer incidences of osteoarthritis per 100 persons attributable to MPK over the ten-year model period.

#### Quality of life

**Fig. 2:**
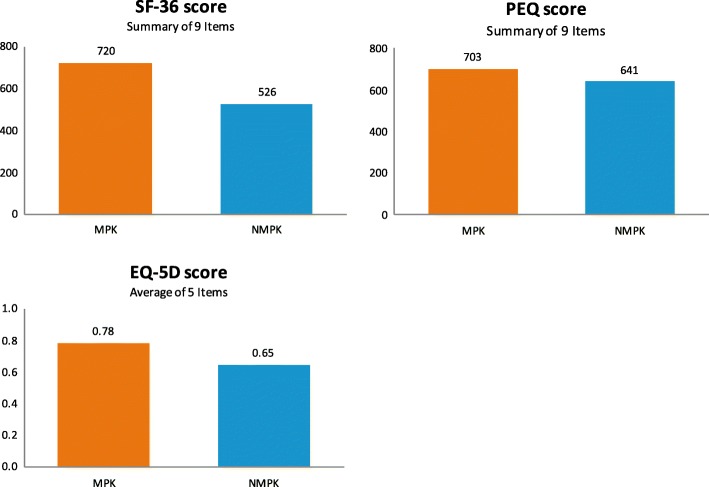
Quality of Life Among MPK and NMPK Users, Note: MPK: microprocessor-controlled knees; NMPK: non-microprocessor controlled knees. Source: [15, 28, 30, 35–40]

On average, subjects experienced a 10% improvement in quality of life when using MPK compared to NMPK, measured by the Prosthesis Evaluation Questionnaire (PEQ) summary score [15, 28, 30, 35, 36]. Seelen [37] reports a 37% higher score in the 36-Item Short Form Health Survey (SF-36) in all amputees as well as recent amputees when they wore MPK compared to NMPK (Fig. 2). The EuroQol five dimensions questionnaire (EQ-5D) scores converted from SF-36 were 0.92 and 0.71 for MPK and NMPK users, respectively, which is consistent with the literature where the MPK group scored 21% higher in EQ-5D than the NMPK group [38–40].

According to the simulation results, the total number of life years in MPK users is 8.8 years greater than in NMPK users (554.4 vs. 545.7) if we observed 100 MPK users and 100 NMPK users over 10 years. Adjusting for quality of life, this leads to 91.4 more QALYs in MPK users compared to NMPK users (453.3 vs. 361.9). The probabilistic sensitivity analysis supports the same conclusions: on average, the number of life years increases by14 years, ranging from 5 to 25 years per 100 MPK users, and the discounted QALYs gained average 102 years, ranging from 82 to 125 years per 100 MPK users.

### Economic benefits

#### Direct healthcare cost and indirect cost

**Fig. 3:**
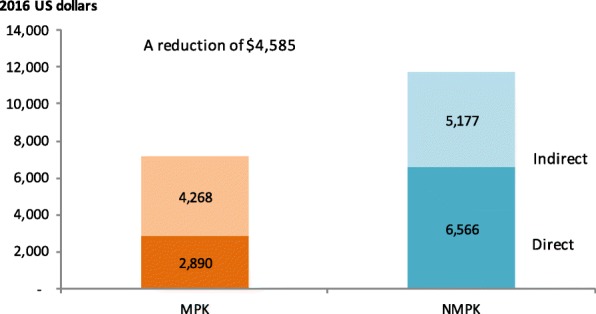
Savings Derived From the Use of MPK in Direct Healthcare Cost and Indirect Cost, Note: MPK: microprocessor-controlled knees; NMPK: non-microprocessor controlled knees. Results are reported on a per person per year basis. All costs are in 2016 U.S. dollars

The simulation for a 10-year period shows reductions in falls and incident osteoarthritis of the intact knees correspond to savings in direct healthcare cost of $3496 and $180 per person per year, respectively. Overall, on a per person per year basis, MPK users have a lower direct healthcare cost than NMPK users, $2890 vs. $6566 (Fig. 3). Moreover, MPK is associated with a reduction of $909 ($4268 vs. $5177) in indirect cost, which includes lost wages, caregiving expenses, and transportation expenses.

#### Cost of device acquisition

Over a 10-year time period, MPK acquisition and repair cost amounts to $7925 per person per year taking into account the effect of survival. The estimate varies from $6054 to $8379 in the probabilistic sensitivity analysis. Similarly, on a per person per year basis, the acquisition and repair of NMPK cost $1638, varying from $785 to $2183 according to the probabilistic sensitivity analysis.

#### Total cost

The resulting total cost in the simulation, defined as the sum of direct ($2890 vs. $6566), indirect ($4268 vs. $5177), and device acquisition and repair cost ($7925 vs. $1638), is $15,083 and $13,382 per person per year for MPK and NMPK users, respectively. The total cost estimates for both MPK and NMPK users are sensitive to the proportion of medical falls among all falls, the average number of falls per faller per year, medical cost per major or minor injury fall, osteoarthritis-related medical cost, and discount rate, as indicated in the one-way sensitivity analyses. In the best scenario, the total cost per person per year for MPK users is $5042 lower than that of NMPK users; in the worst scenario, MPK users cost $5268 more per person per year compared to NMPK users.

In the K1 and K2 population, MPK is associated with a reduction of $4237 per person per year in direct cost and $928 in indirect cost. The total cost associated with MPK is $2022 higher per person per year compared to NMPK. In the best scenario, the total cost of MPK is $5671 less, and in the worst scenario, $6074 more expensive than NMPK.

### Combining economic and clinical benefits

**Fig. 4:**
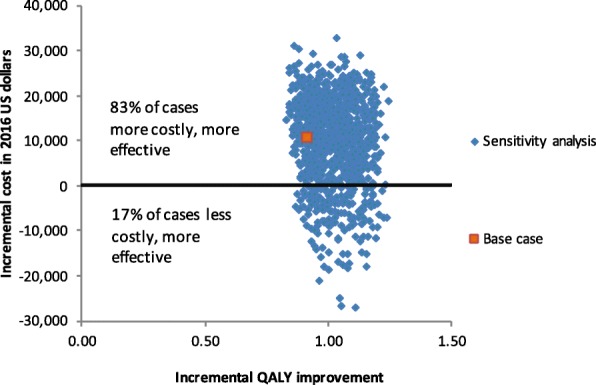
Incremental Cost and Effectiveness of MPK in Comparison to NMPK in K3/K4 Amputees, Note: MPK: microprocessor-controlled knees; NMPK: non-microprocessor controlled knees. QALY: quality adjusted life year. All costs are in 2016 U.S. dollars

When the base case input values were used, for a 10-year time period, MPK resulted in an increase of 0.91 QALY per person and an increase of $10,604 in total cost per person, as illustrated by the orange-red dot in Fig. 4. The corresponding base case ICER is $11,606 per QALY. The blue dots in Fig. 4 were generated from the probabilistic sensitivity analysis. In summary, MPK devices are more effective in all of the simulated scenarios, but also more costly in 83% of the simulated scenarios. The probabilistic sensitivity analysis results in ICERs ranging from -$25,355 to $36,357 per QALY.

In the K1 and K2 population, MPK has an ICER of $13,568 per QALY. MPK may dominate NMPK as suggested in the probabilistic analysis, with an ICER of -$28,302 per QALY, meaning that it incurs lower total cost while leads to higher health status than does NMPK. The highest ICER is $41,498 per QALY in the probabilistic sensitivity analysis.

## Discussion

This study is the first of its kind in the prosthetics literature in the U.S. that integrates both clinical and economic data to assess the cost-effectiveness of advanced prosthetics for transfemoral amputees, specifically MPK. The results suggest that MPK is associated with substantial clinical benefits and cost-effectiveness compared to NMPK. It has been consistently demonstrated in the published literature that MPK leads to clinical improvements, such as improved walking speed, gait symmetry, and obstacle assessment. These clinical improvements, in turn, are associated with sizeable reductions in injurious falls and incident osteoarthritis and as a result, lower mortality rates.

The substantial clinical benefits of MPK can be largely attributed to reductions of falls with injuries and osteoarthritis incidences. This is plausible from a clinical perspective. For example, the computer software in MPK allows for the knee to dynamically adjust to uneven terrain, leading to improved stability and user confidence. The increased stability is thought to reduce cognitive burden and energy expenditure [41]. Increased stability, improved user confidence, and reduced cognitive burden and energy expenditures could all contribute to reductions in the risk of falls [15, 42].

The reduction in the knee moment associated with MPK likely eases the burden on the intact limb and therefore, reduces the probability of developing osteoarthritis in the intact knee. The knee moment is a surrogate for the force an individual absorbs when striking the ground during walking. Because of MPK’s ability to adjust dynamically to uneven ground, MPK users are exposed to weaker forces when striking the ground, or smaller knee moment, than NMPK users. Due to the need for compensation, such forces are absorbed by the lower limb joints of the intact limb, and increase the burden on the healthy knee, hip and ankle, which is the expected mechanism through which osteoarthritis develops in the healthy limb [43].

When examining the life years gained, on average, an MPK user lives 0.09 year longer than an NMPK user over a 10-year time period; but when life years are adjusted by quality, an MPK user gains 0.91 QALYs over an NMPK user. The difference between the number of life years gained and the number of QALYs gained is attributed to the substantial improvement in the quality of life, ranging from 10% to 37%. This finding is consistent with prior evidence that MPK is associated with improved mobility, safety, user confidence, activities of daily living, the ability of living independently, and satisfaction [44–48], and thus substantially the better quality of life for amputees.

While there are some cases where a medical innovation leads to net cost savings, a majority of medical innovations would result in a positive ICER, where a new technology leads to better health but costs more than conventional technologies. The commonly accepted thresholds of fiscal costs for value varies from $50,000 to $150,000 [49–51]. In other words, if a new technology increases the cost by $50,000 or less for every QALY gained, it is considered having good value for the money. MPK has an ICER of $11,606 per QALY, well below the commonly accepted threshold, and thus provides good value for the money. In addition, even when compared to technologies commonly reimbursed by payers in the U.S., MPK fares better. For example, total knee arthroplasty and prophylactic cardioverter defibrillator implantation have an ICER of $14,572 and $76,396 per QALY, respectively [52–56], both of which are higher than that of MPK.

Because the payment system lags behind the advancement in technologies and focuses on cost-cutting rather than value, there is a need to shift the dialogue from a cost-driven payment approach to a value-based payment approach. And this is exactly where the U.S. health care system is headed [57]. However, sophisticated payment approaches such as outcome-based contracts or risk-sharing arrangements require the industry to develop sophisticated methodologies and robust evidence for the economic value of new technologies. As reflected in AOPA’s Prosthetics 2020 Initiative, the industry is aiming to build the infrastructure needed for evidence generation, such as establishing patient registries and collecting clinical and economic data [58]. The initiative will help facilitate such a transition, while this analysis and the research gaps identified could serve as a good starting point.

### Limitations

There is a limited number of studies that directly compare MPK to NMPK, and some model parameters had to come from studies examining a non-amputee population. For example, the proportion of medical falls out of all falls came from the non-amputee literature. The model assumes that parameters are generalizable from a non-amputee population to an amputee population.

The quality of the studies used to extract model parameters is suboptimal. For the parameters needed in the model, there are no published randomized clinical trials that compare MPK to NMPK. In addition, studies cited often have small sample sizes that could lead to large uncertainty in the estimates of MPK’s impact [26, 59]. Also, studies comparing MPK to NMPK often collected data for a limited time period varying from several weeks to a year. For the studies with less than one-year observation period, findings beyond the study period had to be extrapolated for modeling purposes. No studies have examined long-term health outcomes such as obesity, diabetes, and cardiovascular diseases. The lack of studies on these long-term outcomes could potentially lead to an under-estimation of the economic impact of MPK.

Existing studies also have a narrow focus in terms of the amputee population, with an average age of between 38 and 62 and a functional level of K3 or K4. As a result, the effects of MPK on various outcomes might not be generalizable to the Medicare population although the Medicare population does contain numerous younger constituents, for example, dual-eligible combat injured Veterans and others. For the same reason, the modeling results for the K1 and K2 population may not be reliable because most model parameters were extracted from studies for K3 and K4 amputees.

No prior studies examined directly MPK’s impact on incident osteoarthritis and the model relies on expert opinion. While it is generally accepted that differences in gait mechanics manifest in the development of osteoarthritis, there are no studies that demonstrate the causality. Expert consultation suggested that knee moments may represent a reasonable surrogate for the development of osteoarthritis; however, in the absence of long-term studies, it is a limitation.

Finally, the current Medicare payments were used as the numerator of the ICER. Since payment levels are different from the cost of manufacturing MPKs or NMPKs, the estimated numerators of ICERs may not represent the true cost differences between MPKs and NMPKs. If payment levels of MPK and NMPK change in the future, ICER ratios will change accordingly.

## Conclusions

Prior studies have demonstrated that for transfemoral amputees, MPK is superior to NMPK in improving physical function and is associated with sizeable reductions in injurious falls and incident osteoarthritis in the intact limb. Once converted to economic benefits, MPK has an ICER of $11,606 per QALY gained and therefore provides good value for the money compared to NMPK. MPK’s economic benefits are comparable to or even greater than widely reimbursed technologies such as total knee replacement and implantable cardioverter defibrillator.

## Additional file

Additional file 1: **Table S1.** Baseline Characteristics of Medicare Patients with a Unilateral Transfemoral Amputation, 2011–2014. (DOCX 50 kb)

## Abbreviations

AOPA: American orthotic and prosthetic association
CMS: Centers for medicare and medicaid services
EQ-5D: EuroQol five dimensions questionnaire
ICER: Incremental cost-effectiveness ratio
K1: Medicare functional classification level 1
K2: Medicare functional classification level 2
K3: Medicare functional classification level 3
K4: Medicare functional classification level 4
MPK: Microprocessor-controlled prosthetic knee
NMPK: Non–microprocessor-controlled prosthetic knee
PEQ: Prosthesis Evaluation Questionnaire
QALY: Quality-adjusted life year
SF-36: 36-Item Short Form Health Survey

## References

[1] Ziegler-Graham K, MacKenzie EJ, Ephraim PL, Travison TG, Brookmeyer R (2008). Estimating the prevalence of limb loss in the United States: 2005 to 2050. Arch Phys Med Rehabil.

[2] Centers for Disease Control and Prevention (2015). Limb Loss Awareness.

[3] National Center for Health Statistics (2004). Health, United States. 2004: with chartbook on trends in the health of Americans.

[4] Adams PF, Hendershot GE, Marano MA (1996). Current estimates from the National Health Interview Survey. Vital Health Stat 10.

[5] Remes L, Isoaho R, Vahlberg T, Hiekkanen H, Korhonen K, Viitanen M, Rautava P (2008). Major lower extremity amputation in elderly patients with peripheral arterial disease: incidence and survival rates. Aging Clin Exp Res.

[6] Dillingham TR, Pezzin LE (2005). Postacute care services use for dysvascular amputees: a population-based study of Massachusetts. Am J Phys Med Rehabil.

[7] Traugh G, Corcoran P, Reyes R (1975). Energy expenditure of ambulation in patients with above-knee amputations. Arch Phys Med Rehabil.

[8] Gjovaag T, Starholm IM, Mirtaheri P, Hegge FW, Skjetne K (2014). Assessment of aerobic capacity and walking economy of unilateral transfemoral amputees. Prosthetics Orthot Int.

[9] Starholm IM, Mirtaheri P, Kapetanovic N, Versto T, Skyttemyr G, Westby FT, Gjovaag T (2016). Energy expenditure of transfemoral amputees during floor and treadmill walking with different speeds. Prosthetics Orthot Int.

[10] Russell Esposito E, Rabago CA, Wilken J. The influence of traumatic transfemoral amputation on metabolic cost across walking speeds. Prosthetics Orthot Int. 2017; 10.1177/0309364617708649. 10.1177/0309364617708649 28655287

[11] Seymour R, Engbretson B, Kott K, Ordway N, Brooks G, Crannell J, Hickernell E, Wheeler K (2007). Comparison between the C-leg® microprocessor-controlled prosthetic knee and non-microprocessor control prosthetic knees: a preliminary study of energy expenditure, obstacle course performance, and quality of life survey. Prosthetics Orthot Int.

[12] Bellmann M, Schmalz T, Blumentritt S (2010). Comparative biomechanical analysis of current microprocessor-controlled prosthetic knee joints. Arch Phys Med Rehabil.

[13] Kaufman KR, Frittoli S, Frigo CA (2012). Gait asymmetry of transfemoral amputees using mechanical and microprocessor-controlled prosthetic knees. Clin Biomech.

[14] Gailey RS, Roach KE, Applegate EB, Cho B, Cunniffe B, Licht S, Maguire M, Nash MS (2002). The amputee mobility predictor: an instrument to assess determinants of the lower-limb amputee's ability to ambulate. Arch Phys Med Rehabil.

[15] Hafner BJ, Smith DG (2009). Differences in function and safety between Medicare functional classification Level-2 and -3 transfemoral amputees and influence of prosthetic knee joint control. J Rehabil Res Dev.

[16] Rice T, Matsuoka KY (2003). The Impact of Cost-Sharing on Appropriate Utilization and Health Status: A Review of the Literature on Seniors (2004). Med Care Res Rev.

[17] AOPA (2015). Breaking News: DME MACs Issue Draft Policy Revision for Lower Limb Prostheses.

[18] Bureau of Labor Statistics (2017). Consumer Price Index - All Items & Medical Care (2000–2016). United States Department of Labor, Bureau of Labor Statistics.

[19] OANDA Corporation (2017). Average Exchange Rates. OANDA.

[20] Medicare Payment Advisory Commission (2010). Medicare payment advisory commission report to the congress, march 2010. J Pain Palliat Care Pharmacother.

[21] Hazen G (2011). Cohort decomposition for Markov cost-effectiveness models. Med Decis Mak.

[22] Sonnenberg FA, Beck JR (1993). Markov models in medical decision making: a practical guide. Med Decis Mak.

[23] Weinstein MC, O'brien B, Hornberger J, Jackson J, Johannesson M, McCabe C, Luce BR (2003). Principles of good practice for decision analytic modeling in health-care evaluation: report of the ISPOR task force on good research practices—modeling studies. Value Health.

[24] Orendurff MS, Segal AD, Klute GK, McDowell ML, Pecoraro JA, Czerniecki JM (2006). Gait efficiency using the C-leg. J Rehabil Res Dev.

[25] Segal AD, Orendurff MS, Klute GK, McDowell ML, Pecoraro JA, Shofer J, Czerniecki JM (2006). Kinematic and kinetic comparisons of transfemoral amputee gait using C-LEG and MAUCH SNS prosthetic knees. J Rehabil Res Dev.

[26] Kahle JT, Highsmith MJ, Hubbard SL (2008). Comparison of nonmicroprocessor knee mechanism versus C-leg on prosthesis evaluation questionnaire, stumbles, falls, walking tests, stair descent, and knee preference. J Rehabil Res Dev.

[27] Morgenroth DC, Gellhorn AC, Suri P (2012). Osteoarthritis in the disabled population: a mechanical perspective. PM&R.

[28] Hafner BJ, Willingham LL, Buell NC, Allyn KJ, Smith DG (2007). Evaluation of function, performance, and preference as transfemoral amputees transition from mechanical to microprocessor control of the prosthetic knee. Arch Phys Med Rehabil.

[29] Schmalz T, Blumentritt S, Jarasch R (2002). Energy expenditure and biomechanical characteristics of lower limb amputee gait: the influence of prosthetic alignment and different prosthetic components. Gait Posture.

[30] Kaufman KR, Levine JA, Brey RH, McCrady SK, Padgett DJ, Joyner MJ (2008). Energy expenditure and activity of transfemoral amputees using mechanical and microprocessor-controlled prosthetic knees. Arch Phys Med Rehabil.

[31] Datta D, Heller B, Howitt J (2005). A comparative evaluation of oxygen consumption and gait pattern in amputees using intelligent prostheses and conventionally damped knee swing-phase control. Clin Rehabil.

[32] Johansson JL, Sherrill DM, Riley PO, Bonato P, Herr H (2005). A clinical comparison of variable-damping and mechanically passive prosthetic knee devices. Am J Phys Med Rehabil.

[33] Klute GK, Berge JS, Orendurff MS, Williams RM, Czerniecki JM (2006). Prosthetic intervention effects on activity of lower-extremity amputees. Arch Phys Med Rehabil.

[34] Kaufman KR, Levine JA, Brey RH, Iverson BK, McCrady SK, Padgett DJ, Joyner MJ (2007). Gait and balance of transfemoral amputees using passive mechanical and microprocessor-controlled prosthetic knees. Gait Posture.

[35] Prinsen EC, Nederhand MJ, Olsman J, Rietman JS (2015). Influence of a user-adaptive prosthetic knee on quality of life, balance confidence, and measures of mobility: a randomised cross-over trial. Clin Rehabil.

[36] William D, Beasley E, Shaw A (2013). Investigation of the quality of life of persons with a Transfemoral amputation who use a C-leg® prosthetic device. Journal of Prosthetics & Orthotics (JPO).

[37] Seelen HAM, Hemmen B, Schmeets AJ, Ament AJH, Evers SMA (2009). Costs and consequences of a prosthesis with an electronically stance and swing phase controlled knee joint. Technol Disabil.

[38] Brodtkorb T-H, Henriksson M, Johannesen-Munk K, Thidell F (2008). Cost-effectiveness of C-leg compared with non–microprocessor-controlled knees: a modeling approach. Arch Phys Med Rehabil.

[39] Gerzeli S, Torbica A, Fattore G (2009). Cost utility analysis of knee prosthesis with complete microprocessor control (C-leg) compared with mechanical technology in trans-femoral amputees. Eur J Health Econ.

[40] Cutti AG, Lettieri E, Del Maestro M, Radaelli G, Luchetti M, Verni G, Masella C (2017). Stratified cost-utility analysis of C-leg versus mechanical knees: findings from an Italian sample of transfemoral amputees. Prosthetics Orthot Int.

[41] Highsmith MJ, Kahle JT, Bongiorni DR, Sutton BS, Groer S, Kaufman KR (2010). Safety, energy efficiency, and cost efficacy of the C-leg for transfemoral amputees: a review of the literature. Prosthetics Orthot Int.

[42] Hafner BJ, Askew RL (2015). Physical performance and self-report outcomes associated with use of passive, adaptive, and active prosthetic knees in persons with unilateral, transfemoral amputation: randomized crossover trial. J Rehabil Res Dev.

[43] Felson DT (2013). Osteoarthritis as a disease of mechanics. Osteoarthr Cartil.

[44] Berry D, Olson MD, Larntz K (2009). Perceived stability, function, and satisfaction among transfemoral amputees using microprocessor and nonmicroprocessor controlled prosthetic knees: a multicenter survey. J Prosthet Orthot.

[45] Theeven P, Hemmen B, Rings F, Meys G, Brink P, Smeets R, Seelen H (2011). Functional added value of microprocessor-controlled knee joints in daily life performance of Medicare functional classification Level-2 amputees. Scand J Rehabil Med.

[46] Theeven PJ, Hemmen B, Geers RP, Smeets RJ, Brink PR, Seelen HA (2012). Influence of advanced prosthetic knee joints on perceived performance and everyday life activity level of low-functional persons with a transfemoral amputation or knee disarticulation. J Rehabil Med.

[47] Sawers AB, Hafner BJ (2013). Outcomes associated with the use of microprocessor-controlled prosthetic knees among individuals with unilateral transfemoral limb loss: a systematic review. J Rehabil Res Dev.

[48] Kannenberg A, Zacharias B, Probsting E (2014). Benefits of microprocessor-controlled prosthetic knees to limited community ambulators: systematic review. J Rehabil Res Dev.

[49] Institute for Clinical and Economic Review (2017). Final Value Assessment Framework: Updates for 2017–2019.

[50] Weinstein MC (2008). How much are Americans willing to pay for a quality-adjusted life year?. Med Care.

[51] Neumann PJ, Cohen JT, Weinstein MC (2014). Updating cost-effectiveness--the curious resilience of the $50,000-per-QALY threshold. N Engl J Med.

[52] Losina E, Walensky RP, Kessler CL, Emrani PS, Reichmann WM, Wright EA, Holt HL, Solomon DH, Yelin E, Paltiel AD (2009). Cost-effectiveness of total knee arthroplasty in the United States: patient risk and hospital volume. Arch Intern Med.

[53] Waimann CA, Fernandez-Mazarambroz RJ, Cantor SB, Lopez-Olivo MA, Zhang H, Landon GC, Siff SJ, Suarez-Almazor ME (2014). Cost-effectiveness of total knee replacement: a prospective cohort study. Arthritis Care Res.

[54] Ruiz D, Koenig L, Dall TM, Gallo P, Narzikul A, Parvizi J, Tongue J (2013). The direct and indirect costs to society of treatment for end-stage knee osteoarthritis. J Bone Joint Surg Am.

[55] Elmallah RK, Chughtai M, Khlopas A, Bhowmik-Stoker M, Bozic KJ, Kurtz SM, Mont MA (2017). Determining cost-effectiveness of total hip and knee arthroplasty using the short form-6D utility measure. J Arthroplast.

[56] Garcia-Perez L, Pinilla-Dominguez P, Garcia-Quintana A, Caballero-Dorta E, Garcia-Garcia FJ, Linertova R, Imaz-Iglesia I (2015). Economic evaluations of implantable cardioverter defibrillators: a systematic review. Eur J Health Econ.

[57] Centers for Medicare and Medicaid Services (2016). Better care. Smarter spending. Healthier people: improving quality and paying for what works. CMS Fact Sheet Accessed.

[58] The American Orthotic and Prosthetic Association (2017). Prosthetics 2020 and Orthotics 2020.

[59] Wong CK, Rheinstein J, Stern MA (2015). Benefits for adults with Transfemoral amputations and peripheral artery disease using microprocessor compared with nonmicroprocessor prosthetic knees. Am J Phys Med Rehabil.

[60] Dederer L. Quality of life of amputee patients after supply with the electronically controlled knee pass part “C-leg”: Prospective consultation of patients with care. Dissertation (in German): Westfälische Wilhelms-Universität Münster, Germany; 2013.

[61] Kelsey JL, Berry SD, Procter-Gray E, Quach L, Nguyen US, Li W, Kiel DP, Lipsitz LA, Hannan MT (2010). Indoor and outdoor falls in older adults are different: the maintenance of balance, independent living, intellect, and zest in the elderly of Boston study. J Am Geriatr Soc.

[62] Schiller JS, Kramarow EA, Dey AN. Fall injury episodes among noninstitutionalized older adults: United States, 2001-2003. Adv Data. 2007;21(392):1–16. https://www.ncbi.nlm.nih.gov/pubmed/17953135. 17953135

[63] Verma SK, Willetts JL, Corns HL, Marucci-Wellman HR, Lombardi DA, Courtney TK (2016). Falls and fall-related injuries among community-dwelling adults in the United States. PLoS One.

[64] Sterling DA, O'Connor JA, Bonadies J (2001). Geriatric falls: injury severity is high and disproportionate to mechanism. J Trauma.

[65] Kim SB, Zingmond DS, Keeler EB, Jennings LA, Wenger NS, Reuben DB, Ganz DA (2016). Development of an algorithm to identify fall-related injuries and costs in Medicare data. Inj Epidemiol.

[66] Mundell B, Maradit Kremers H, Visscher S, Hoppe K, Kaufman K. Direct medical costs of accidental falls for adults with transfemoral amputations. Prosthetics Orthot Int. 2017; 10.1177/0309364617704804. 10.1177/0309364617704804 28641476

[67] Miller WC, Deathe AB, Speechley M, Koval J (2001). The influence of falling, fear of falling, and balance confidence on prosthetic mobility and social activity among individuals with a lower extremity amputation. Arch Phys Med Rehabil.

[68] Burns ER, Stevens JA, Lee R (2016). The direct costs of fatal and non-fatal falls among older adults - United States. J Saf Res.

[69] Stevens JA, Corso PS, Finkelstein EA, Miller TR (2006). The costs of fatal and non-fatal falls among older adults. Injury Prev.

[70] Pension Rights Center. Sources for income of older adults: US Department of Labor, Bureau of Labor Statistics; 2016.

[71] Bureau of Labor Statistics (2015). May 2015 National Occupational Employment and Wage Estimates.

[72] Bureau of Labor Statistics (2015). Labor Force Statistics from the Current Population Survey - Employment status of the civilian noninstitutional population by age, sex, and race.

[73] Xie F, Kovic B, Jin X, He X, Wang M, Silvestre C (2016). Economic and humanistic burden of osteoarthritis: a systematic review of large sample studies. PharmacoEconomics.

[74] Berger A, Hartrick C, Edelsberg J, Sadosky A, Oster G (2011). Direct and indirect economic costs among private-sector employees with osteoarthritis. J Occup Environ Med.

[75] Dibonaventura M, Gupta S, McDonald M, Sadosky A (2011). Evaluating the health and economic impact of osteoarthritis pain in the workforce: results from the National Health and wellness survey. BMC Musculoskelet Disord.

[76] Sanders GD, Neumann PJ, Basu A, Brock DW, Feeny D, Krahn M, Kuntz KM, Meltzer DO, Owens DK, Prosser LA (2016). Recommendations for conduct, methodological practices, and reporting of cost-effectiveness analyses: second panel on cost-effectiveness in health and medicine. JAMA.

