# Supplementary material for: Economic benefits of microprocessor controlled prosthetic knees: a modeling study
Source: J Neuroeng Rehabil. 2018 Sep 5;15(Suppl 1):62. doi: 10.1186/s12984-018-0405-8 (PMC6157253; doi:10.1186/s12984-018-0405-8)
Supplement: Supplementary file 1 — Table S1. Baseline Characteristics of Medicare Patients with a Unilateral Transfemoral Amputation, 2011–2014. (DOCX 50 kb) [file 12984_2018_405_MOESM1_ESM.docx]

Table S1. Baseline Characteristics of Medicare Patients with a Unilateral Transfemoral Amputation, 2011-2014

| **Characteristics** | **K1/2 Patients** | **K3/4 Patients** | |
| --- | --- | --- | --- |
|  | **Non-MPK Prosthetics** | **MPK Prosthetics** | **Non-MPK Prosthetics** |
| CPT codes | Presence of L5321 but not the CPT codes for MPK | Presence of L5321 and any of the following codes: L5856, L5857, L5858, L5859 | Presence of L5321 but not the CPT codes for MPK |
| Number of patients | 878 | 549 | 348 |
| Age |  |  |  |
| Mean | 72.5 | 65.0 | 66.2 |
| Standard Deviation | 11.1 | 12.3 | 11.9 |
| 1st Percentile | 47 | 29 | 30 |
| 25th Percentile | 66 | 58 | 59 |
| 50th Percentile (Median) | 72 | 67 | 67 |
| 75th Percentile | 81 | 73 | 73 |
| 99th Percentile | 94 | 88 | 92 |
| Gender |  |  |  |
| Female (%) | 44.8% | 25.7% | 33.9% |
| Race |  |  |  |
| White (%) | 59.0% | 77.6% | 57.8% |
| Black (%) | 28.8% | 16.4% | 32.8% |
| Hispanic and Others (%) | 12.2% | 6.0% | 9.5% |
| All cause mortality |  |  |  |
| Death within 1 year of device fitting (%) | 20.2% | 6.0% | 13.5% |
| Death within 2 years of device fitting (%) | 32.8% | 12.2% | 26.1% |
| Etiology |  |  |  |
| Trauma (%) | 41.3% | 37.5% | 39.1% |
| Vascular disease (%) | 56.8% | 56.5% | 56.9% |
| Cancer (%) | * | 2.7% | * |
| Other (%) | * | 3.3% | * |
| Chronic conditions |  |  |  |
| Obesity | 5.9% | 6.4% | 4.9% |
| Diabetes | 61.8% | 45.2% | 54.6% |
| Rheumatoid Arthritis / Osteoarthritis | 45.8% | 41.7% | 39.4% |
| Low back pain | 28.2% | 29.5% | 30.5% |
| Depression | 29.2% | 24.8% | 25.6% |
| Acute Myocardial infarction | 5.5% | 3.1% | 6.0% |
| Ischemic Heart Disease | 69.5% | 58.8% | 62.1% |
| Stroke / Transient Ischemic Attack | 13.9% | 7.5% | 9.5% |
| Chronic Obstructive Pulmonary Disease | 35.4% | 27.7% | 37.9% |
| Atrial Fibrillation | 17.7% | 12.2% | 12.9% |
| Heart Failure | 48.3% | 25.7% | 36.2% |
| Hypertension | 88.7% | 78.3% | 83.3% |
| Osteoarthritis (Hip) | 9.1% | 8.4% | 8.3% |
| Osteoarthritis (Knee) | 16.3% | 20.6% | 16.7% |

Note: MPK: microprocessor-controlled knees; NMPK: non-microprocessor controlled knees. CPT: Current Procedure Terminology. K1-K4: Medicare Functional Classification Level 1 to 4, respectively. The analysis included all patients who had a unilateral transfemoral amputation and received a prosthetic device during 2012-2013, allowing 12 months of observation period pre- and post-device fitting for baseline and follow-up, respectively. There were a total of 2,635 Medicare beneficiaries included in the analysis, with 860 of them having a missing K Level classification. After 2011, only K3 or K4 patients were eligible for MPK under Medicare reimbursement rules. The mortality rate for K1/2 and K3/4 patients is18.0% and 9.3% respectively and the knee osteoarthritis prevalence is 16.3% and 19.1% respectively. Columns marked "Data not available" and cells with "*" indicate device categories and cells with fewer than 11 observations (including zero) or are otherwise blinded for HIPAA compliance.
